# Pandemic Deployment of a Smarthealth Technology to Improve Stress in Dementia Family Caregivers

**DOI:** 10.1093/geroni/igab046.1737

**Published:** 2021-12-17

**Authors:** Karen Rose, Kristina Gordon, Emma Schlegel, Matthew McCall, Ye Gao, Jason Jabbour, Eunjung Ko

**Affiliations:** 1 The Ohio State University, Columbus, Ohio, United States; 2 The University of Tennessee Department Of Psychology, Knoxville, Tennessee, United States; 3 The Ohio State University College Of Nursing, Columbus, Ohio, United States; 4 University Of Virginia Department of Computer Science, Charlotesville, Virginia, United States; 5 University of Virginia Department of Engineering Systems and Environment, Charlotesville, Virginia, United States

## Abstract

Caregiving stress from repetitive and heavy caregiving workloads can trigger poor emotional health, such as stress, anxiety, and depression, leading to higher caregiver mortality rates. Interest in technology-based interventions for this population has increased among researchers due to availability, acceptability, and flexibility compared to in-person services, especially now, during an unprecedented pandemic. Our study focuses on in-home SmartHealth technologies for caregivers of persons with Alzheimer’s Disease and related dementias, delivered using Ecological Momentary Assessment and a novel acoustic monitoring, mood recognition, and self-learning recommendation system. The system provides mindfulness-based stress management in response to interpersonal conflict in real-time. We will report challenges and solutions of creating and deploying a SmartHealth system for older adults in their home during the COVID-19 pandemic. Potential effects of this system on caregivers' emotional health are also examined. Findings suggest SmartHealth technologies may assist caregiving populations adapt and thrive in a new, more isolated normal.

